# Effect of a 12-Week Polyphenol Rutin Intervention on Markers of Pancreatic β-Cell Function and Gut Microbiota in Adults with Overweight without Diabetes

**DOI:** 10.3390/nu15153360

**Published:** 2023-07-28

**Authors:** Akarsh Mathrani, Wilson Yip, Ivana R. Sequeira-Bisson, Daniel Barnett, Oliver Stevenson, Michael W. Taylor, Sally D. Poppitt

**Affiliations:** 1 School of Biological Sciences, University of Auckland, Auckland 1010, New Zealand; amat116@aucklanduni.ac.nz (A.M.); wyip015@aucklanduni.ac.nz (W.Y.); i.sequeira@auckland.ac.nz (I.R.S.-B.); 2 High-Value Nutrition National Science Challenge, Auckland 1010, New Zealand; 3 Human Nutrition Unit, University of Auckland, Auckland 1024, New Zealand; 4 Department of Statistics, University of Auckland, Auckland 1010, New Zealand; dbar344@aucklanduni.ac.nz (D.B.); o.g.stevenson@gmail.com (O.S.); 5 Department of Medicine, University of Auckland, Auckland 1010, New Zealand

**Keywords:** prebiotic polyphenol rutin, pancreas β-cell function, gut microbiota

## Abstract

Supplementation with prebiotic polyphenol rutin is a potential dietary therapy for type 2 diabetes prevention in adults with obesity, based on previous glycaemic improvement in transgenic mouse models. Gut microbiota are hypothesised to underpin these effects. We investigated the effect of rutin supplementation on pancreatic β-cell function measured as C-peptide/glucose ratio, and 16S rRNA gene-based gut microbiota profiles, in a cohort of individuals with overweight plus normoglycaemia or prediabetes. Eighty-seven participants were enrolled, aged 18–65 years with BMI of 23–35 kg/m^2^. This was a 12-week double-blind randomised controlled trial (RCT), with 3 treatments comprising (i) placebo control, (ii) 500 mg/day encapsulated rutin, and (iii) 500 mg/day rutin-supplemented yoghurt. A 2-h oral glucose tolerance test (OGTT) was performed at baseline and at the end of the trial, with faecal samples also collected. Compliance with treatment was high (~90%), but rutin in both capsule and dietary format did not alter pancreatic β-cell response to OGTT over 12 weeks. Gut bacterial community composition also did not significantly change, with *Firmicutes* dominating irrespective of treatment. Fasting plasma glucose negatively correlated with the abundance of the butyrate producer *Roseburia inulinivorans*, known for its anti-inflammatory capacity. This is the first RCT to investigate postprandial pancreatic β-cell function in response to rutin supplementation.

## 1. Introduction

Type 2 diabetes (T2D) is a major global health concern, with prevalence increasing rapidly [1,2,3]. In 2019 there were >400 million cases worldwide [2,4], with ~1.6 million deaths directly attributed to this disease each year [4]. Overweight and obesity are associated with insulin resistance, hyperinsulinaemia, and dysglycaemia and are major risk factors for developing T2D [5,6]. The peptide hormone amylin is co-secreted from the pancreas at equimolar ratios with insulin [7,8], with obesity also leading to hyperamylinaemia [9,10]. Importantly, at high concentrations human amylin has been shown to misfold and form β-sheet fibrils which may become toxic to the pancreatic β-cells, in turn suppressing insulin secretion [11,12,13,14]. Amylin β-sheet fibrils or amyloid deposits have been observed within the pancreas of >90% of T2D patients [15,16,17]. Notably, however, suppression of β-sheet fibrillation can decrease β-cell toxicity and deposition of amyloid within pancreatic tissue [18,19], suggesting a window of opportunity by which endogenous insulin production may be preserved. Dietary polyphenols have been proposed as a therapeutic avenue for the prevention of amyloid formation [20,21,22,23,24].

Polyphenols are commonly found in fruits and vegetables, with their potential role in preventing and managing T2D well recognised [25,26]. Antioxidant properties have previously been implicated, but the recent focus has been on preservation of pancreatic β-cell function [20]. Flavonoids, a subclass of polyphenols, act as small molecule inhibitors that may prevent amyloid formation [22,23,24]. In vitro data show the flavonoids rutin and quercetin to act as potent inhibitors of human amylin aggregation [21]. Rutin is the glycoside of quercetin [27], but unlike quercetin, which is absorbed in the small intestine, rutin undergoes bacterial fermentation and slower absorption in the colon [28,29,30]. Anti-diabetic activity in human amylin transgenic mice has also been reported [21,31]. Rutin as an antioxidant has previously been observed in human trials, administered orally as 500 mg/day over 6–8 weeks [32,33], the dose prescribed in commercial supplements globally. An equivalent dose also ameliorated amyloid deposits in transgenic mice [31], having been shown in human trials to increase circulating rutin and quercetin levels by at least 2.5-fold [32]. To date, no clinical interventions have investigated the effect of rutin on pancreatic β-cell function.

An association of metabolic disorders such as T2D with the human gut microbiota is well established [34,35,36]. Particularly relevant to T2D onset is the saccharolytic fermentation of resistant starch and other indigestible carbohydrates, a significant energy-harvesting mechanism that is mediated by the gut microbiota and provides as much as 10% of the host’s total energy requirements [37,38]. The short chain fatty acid (SCFA) butyrate is of special interest, with T2D patients reported to harbour fewer butyrate-producing bacteria [34,39] and with butyrate linked to decreased adipocyte inflammation [40] and improved insulin sensitivity [41,42] in mouse models. Since rutin is a known prebiotic, gut microbes are likely involved in mediating its influence on host health. A recent in vitro study has established a direct interaction between rutin and human gut bacteria, with members from various families (*Lachnospiraceae*, *Enterobacteriaceae*, *Tannerellaceae, Erysipelotrichaceae*) enriched following rutin inclusion during anaerobic incubation [43].

In this study we aimed to evaluate a 500 mg/day oral rutin treatment on pancreatic β-cell function in adults with obesity, across a range of dysglycaemias, in a 3-treatment randomised controlled trial (RCT) of 12 weeks’ duration. To determine the prebiotic potential of rutin within a food matrix, two delivery formats, comprising encapsulated rutin powder and rutin-enriched dairy yoghurt, were investigated. A 2-h oral glucose tolerance test (OGTT) was used to probe postprandial response of C-peptide/glucose ratio as an indirect measure of pancreatic β-cell function. C-peptide is secreted by β-cells after cleavage of proinsulin [44] and, with low variability and steady hepatic clearance [45], more accurately represents endogenous insulin secretion [46]. We also report gut microbiota profiles over the 3-month dietary intervention.

## 2. Materials and Methods

### 2.1. Study Design and Participant Recruitment

The clinical trial was conducted at the University of Auckland Human Nutrition Unit (HNU) research clinic, Auckland, New Zealand. Study participants were recruited between June 2018 and January 2019 from residential communities in Auckland via advertisement. Eligibility was determined based on these inclusion criteria at screen: Asian Chinese and European Caucasian ethnicity; aged 25 to 70 years; body mass index (BMI) ≥ 23 kg/m^2^; Finnish Diabetes Risk Score (FINDRISC) ≥12 [47,48]; fasting plasma glucose (FPG) 5.6 to 6.9 mmol/L [49]; and stable body weight for the 3 months prior to the trial (<5% change in body mass) with no intention of losing weight during the trial period. Exclusion criteria were smoking, BMI ≥ 40 kg/m^2^, pregnancy, diagnosis of any significant comorbidities such as diabetes or cancer, dislike/unwillingness to consume study food items, allergies to study food items (e.g., rutin allergy, lactose intolerance), medications or supplements that may influence participant glycaemia, and parallel or recent participation in other clinical studies. All participants provided written, informed consent before enrolling into the study, and ethics approval for the trial was granted by the Auckland Regional Health and Disabilities Committee (Reference: 18/CEN/52). The trial was registered with the Australian New Zealand Clinical trial registry (Trial ID: ACTRN12618000656235).

**Figure 1:**
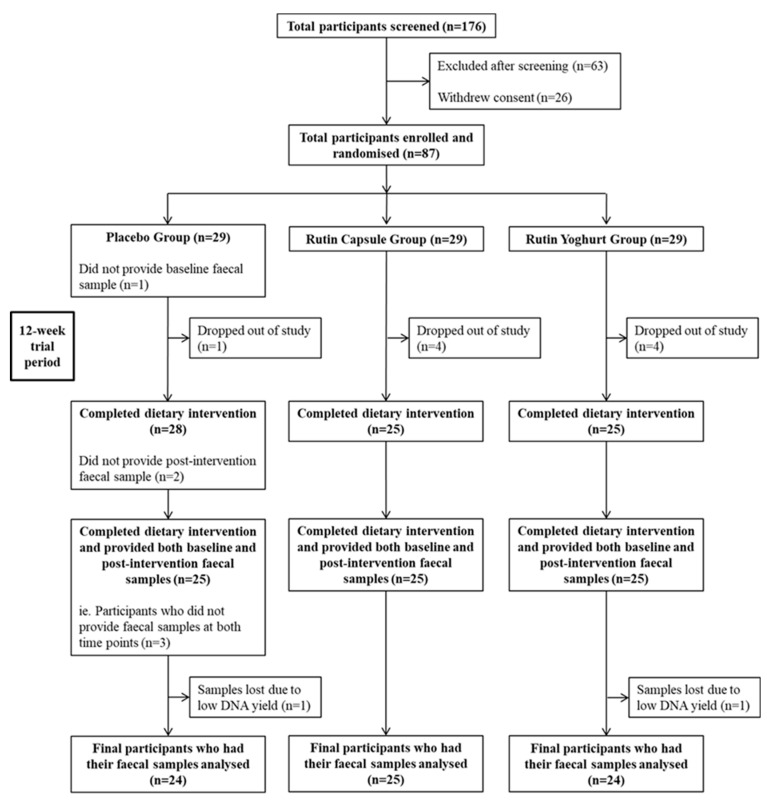
CONSORT flow chart for the rutin dietary intervention trial.

The intervention was a double-blind, randomised, 3-arm study conducted over 12 weeks, in 87 participants. Twenty-nine participants were randomised into each intervention group (Figure 1). Blinding required all participants to consume both capsules and yoghurt products in each treatment. The three treatment groups were: (i) placebo capsule + placebo yoghurt (Control); (ii) 500 mg rutin capsule + placebo yoghurt (rutin capsule, RC); and (iii) 500 mg rutin-enriched yoghurt + placebo capsule (rutin yoghurt, RY). A random number-generating computer program was utilised for randomisation.

Participant demographics are summarised in Table 1, and Supplementary Tables S1–S3. Each participant was required to consume the assigned intervention product once daily over the 12-week period as part of their breakfast meal. Compliance with treatment was assessed by asking the participants to return leftover capsules, and empty yoghurt pottles plus unconsumed yoghurt. Participants were requested to maintain their habitual diet and activity levels for the 3-month trial.

### 2.2. Clinical Intervention Days (CID)

The 3-month intervention comprised 4 clinical intervention days (CIDs) conducted in clinic, namely CID 1 (baseline, 0 weeks), CID 2 (4 weeks), CID 3 (8 weeks), and CID 4 (12 weeks, end of trial). For all CIDs, participants arrived at the Human Nutrition Unit at 8 am after an overnight fast, and anthropometric measurements were recorded and a blood sample was collected. At CID 1 (baseline) and CID 4 (end of trial) an OGTT was then also conducted. Upon arrival, participants were given 250 mL water to drink and a peripheral venous cannula was inserted to facilitate repeat blood sampling. The 2-h standardised OGTT was conducted using a 75 g glucose drink. Blood was collected (5 mL per time point) at baseline (fasting t = 0) and following consumption of the glucose drink, at time points (t = 30, 60, 90, 120 min) into fluoride/oxalate, SST, and K2 EDTA vacutainers respectively. Aliquots were stored at −80 °C until batch analysis. Faecal samples were also collected at CID 1 and CID 4 for gut microbiota composition analysis (see ‘Gut microbiota’ section below for details). At CID 1 body composition was assessed by dual-energy X-ray absorptiometry (DXA) scan, performed at Auckland City Hospital (see ‘Body composition analysis’ section below for details). At all CIDs, adverse events and changes in medications were also recorded, and compliance with treatment products was assessed.

### 2.3. Anthropometry

Body weight was measured using digital scales (Mettler Toledo Spider 2, Zurich, Switzerland). Height was measured using a wall-mounted stadiometer (Seca 222, Hamburg, Germany). Both were measured lightly clad and without shoes. Waist and hip circumference were measured using a non-stretch tape (Abbott Laboratories, Abbott Park, IL, USA). The waist was measured at the midpoint of the lowest palpable rib and the hip joint bone, around the belly. The hip was measured at the widest point over the greater trochanter. Blood pressure was measured using a Dinamap Critikon sphygmomanometer (GE Healthcare, Shanghai, China) on the non-dominant arm when rested and seated. All parameters were measured twice, and the average was reported.

### 2.4. Body Composition

Body composition was assessed by DXA (iDXA, GE Healthcare, Madison, WI, USA). Total body fat %, abdominal fat %, and visceral fat % were calculated according to the formulae below:

$$\mathrm{Total}\;\mathrm{body}\;\mathrm{fat}\;\left(\%\right)=\frac{\mathrm{total}\;\mathrm{body}\;\mathrm{fat}\;\mathrm{mass}\;\left(g\right)}{\mathrm{total}\;\mathrm{body}\;\mathrm{fat}\;\mathrm{mass}\;\left(g\right)+\;\mathrm{total}\;\mathrm{body}\;\mathrm{lean}\;\mathrm{mass}\;\left(g\right)}\times 100$$

$$\mathrm{Abdominal}\;\mathrm{fat}\;\left(\%\right)=\frac{\mathrm{abdominal}\;\mathrm{fat}\;\mathrm{mass}\;\left(g\right)}{\mathrm{abdominal}\;\mathrm{fat}\;\mathrm{mass}\;\left(g\right)+\mathrm{abdominal}\;\mathrm{lean}\;\mathrm{mass}\;\left(g\right)}\times 100$$

$$\mathrm{Visceral}\;\mathrm{fat}\;\left(\%\right)=\frac{\mathrm{visceral}\;\mathrm{fat}\;\mathrm{mass}\;\left(g\right)}{\mathrm{abdominal}\;\mathrm{fat}\;\mathrm{mass}\;\left(g\right)}\times 100$$

### 2.5. Biochemistry

FPG at screening was analysed from whole blood with manual conversion to plasma glucose (Reflotron Plus Desk Top Analyser, Mannheim, Germany) [50]. Biochemical analyses at CID 1 to CID 4 were conducted as batch analyses at the University of Auckland Liggins Institute upon completion of the trial. Glucose, lipids, and liver function tests (LFT) were analysed using a Cobas C311 analyser (Roche Diagnostics, Indianapolis, IN, USA). Glucose was analysed using the hexokinase method from plasma samples [51], total cholesterol (TC) using the cholesterol esterase/cholesterol oxidase/peroxidase method [52], triglyceride (TG) using the lipase/glycerol kinase method [53], and HDL-C using the detergent/cholesterol esterase/cholesterol oxidase/peroxidase method. LDL-cholesterol was calculated using the Friedwald formula (LDL-C (mmol/L) = TC (mmol/L) − HDL-C (mmol/L) − TG (mmol/L)/2.2) [54]. Liver enzymes alanine aminotransferase (ALT) and aspartate aminotransferase (AST) were analysed using the IFCC method [55]; alkaline phosphatase using the IFGG colorimetric assay method [56]; gamma-glutamyl transferase (GGT) using the Szasz method [57]. Insulin and C-peptide were analysed using a Cobas E411 analyser (Roche Diagnostics, Indianapolis, IN, USA) using Elecsys immunoassay with Electrochemiluminescence (ECL) technology. All enrolled participants had impaired FPG (≥5.6–6.9 mmol/L) and were identified as prediabetic at screen. Subsequent analysis of FPG at baseline CID 1 after completion of the trial identified that a substantial proportion had reverted to normoglycamia; hence, study aims were revised to include analysis of the normoglycaemic and prediabetic sub-groups.

### 2.6. Composition of Rutin Products

The capsules were formulated and manufactured by Alpha Laboratories NZ Ltd. (Auckland, New Zealand) following good manufacturing practice (GMP) and under license to manufacture therapeutic goods. Treatment capsules contained 250 mg rutin powder; placebo capsules contained 250 mg maltodextrin. Participants were instructed to take two capsules per day to meet the 500 mg daily dosage. A complete formulation profile is presented in Supplementary Table S4. The dairy yoghurts were formulated by the Science of Food Platform within the National Science Challenge High-Value Nutrition (NSC-HVN) program at the Riddet Institute, Massey University, Palmerston North, NZ. The rutin-enriched yoghurt was manufactured using the ingredient FlavoPlus [58], a proprietary rutin–sodium caseinate co-precipitate developed as part of this research programme, and the placebo yoghurt was manufactured using only sodium caseinate. All other ingredients and nutritional components of the two yoghurts were identical. The fermentation of yoghurts was performed in a commercial dairy factory, following GMP under a Food Safety Environment License number MPI000368/1, utilising a culture composed of *Lactobacillus delbrueckii* (subsp. *bulgaricus*) and *Streptococcus thermophilus* (YF-L811, YoFlex^®^, CHR Hansen Pty. Ltd., Melbourne, Australia). Five hundred units of the culture per 2500 L of whole milk + 2% skim milk powder were combined together and incubated at 85 °C for 30 min. The final product was packaged with a net weight of 190 g per serving, containing 500 mg rutin. A complete formulation profile is presented in Supplementary Table S5. Although both treatments provided rutin at equivalent doses, rutin has low solubility and therefore bioavailability, and hence, the rate of gastrointestinal absorption may differ depending on extent of degradation [59]. In an attempt to compensate for potential differences in rutin delivery, rutin added to yoghurt was microencapsulated with sodium caseinate, a water-soluble compound derived from milk casein, with the intention to protect the polyphenol from degradation and improve its cellular uptake [58].

### 2.7. Gut Microbiota

#### 2.7.1. Faecal Sample Collection

Faecal samples were collected at baseline CID 1 and after the 12-week dietary intervention CID 4 into a sterile collection tube, frozen at home, and then delivered to the research clinic without sample thawing. All faecal samples were then stored at −80 °C prior to DNA extraction.

#### 2.7.2. DNA Extraction

Genomic DNA was extracted from 250 mg of thawed faecal sample using International Human Microbiome Standards (IHMS) Protocol #9 [60], which is a repeated bead-beating method utilising 0.1 mm silica and 3 mm glass beads. Cell lysis was performed using a non-commercial lysis buffer recipe (500 mM NaCl, 50 mM Tris-HCl at pH 8.0, 50 mM EDTA and 4% SDS) as per the protocol, however, a Qiagen Tissuelyser II (Retsch, Boston Industries, Walpole, MA, USA) was used (frequency of 30 Hz, for two cycles of 1.5 min) to break the cells instead of the FastPrep^®^-24 Instrument (116004500, MP Biomedicals, Irvine, CA, USA) with CoolPrep Adapter (6002-528, MP Biomedicals, Irvine, CA, USA) as advised by the protocol. A QIAamp DNA Minikit (Qiagen, 51306, Venlo, The Netherlands) was utilised in the final steps of the protocol for removal of RNA, protein and purification, as recommended by the protocol. Negative DNA extractions containing 250 µL of sterile water were also carried out to test for potential contamination. All extracts were subsequently analysed on a Nanodrop 3300 fluorospectrometer (Nanodrop Technologies Inc., Wilmington, WI, USA) to determine DNA quality and concentration.

#### 2.7.3. 16S rRNA Gene-Targeted PCR and Sequencing

Bacterial community structure was analysed by PCR amplification and then sequencing of the highly variable V3-V4 region of the 16S rRNA gene. KAPA High Fidelity HotStart Readymix PCR Kit (Kapa Biosystems^®^, Wilmington, MA, USA) was utilised for this amplification, with ~50 ng of template genomic DNA used per reaction. Labelled with Illumina MiSeq-compatible adaptors, the primer pair 341F (5′-CCTACGGGNGGCWGCAG-3′) and 785R (5′-GACTACHVGGGTATCTAATCC-3′) [61] was used with the following thermocycling conditions: initial denaturation and activation of enzymes at 95 °C for 3 min, followed by 25 cycles of denaturation (95 °C for 30 s), annealing (55 °C for 30 s) and elongation (72 °C for 30 s), with a final extension of 72 °C for 10 min. PCR products were electrophoresed on 1% (*w*/*v*) agarose gels with SYBR Safe nucleic acid stain (Invitrogen Co., Waltham, MA, USA) to ensure correct amplicon size. Negative PCR controls, in which nuclease-free H_2_O was used instead of template DNA, as well as amplifications of eluates from the negative DNA extractions, did not produce any visible DNA products. Randomly selected negative controls were nonetheless sequenced even if no product was visible on an agarose gel.

Amplified PCR products were further purified using AMPure magnetic beads (Beckman-Coulter Inc., Brea, CA, USA) in accordance with manufacturer instructions, and quantified using the Qubit dsDNA high-sensitivity kit (Invitrogen Co., Carlsbad, NM, USA). DNA concentrations were standardised and submitted to the sequencing provider (Auckland Genomics Ltd., Auckland, New Zealand) for Illumina MiSeq sequencing (2 × 300 bp chemistry).

#### 2.7.4. Bioinformatics

A bioinformatics pipeline [62] compatible with the software package USEARCH [63] was utilised to join the raw pairs of sequence reads, trim away primer-binding regions, quality filter, and study the merged sequences [64]. The sequence reads were further error-corrected and zero-radius operational taxonomic units (zOTUs) of 100% similarity were generated [65]. Non-target (including human mitochondrial) sequences were removed, and the SILVA v123 database was used to assign taxonomy to each zOTU [66,67]. All unassigned sequences were manually checked via BLAST nucleotide search, and any sequences producing non-target (i.e., not bacteria) hits were removed [68]. Sequence data from each sample were rarefied to 1998 reads/sample. All negative control samples sequenced (*n* = 15) produced a negligible number of sequence reads (16.8 ± 14.6) and were summarily discarded during the quality filtration steps of the pipeline. The 16S rRNA gene sequence data were deposited in Genbank under SRA Bioproject PRJNA946170.

### 2.8. Statistical Analyses

#### 2.8.1. Calculation of Power

Power was calculated using SYSTAT 13 (Systat Software Inc., Chicago, IL, USA) [69] and based on mean change (±SD) in the primary endpoint and, incremental area under the curve (iAUC) of C-peptide/glucose (0.53 nmol/mmol ± 0.23), following a standard 75 g OGTT [70]. A sample size of 81 participants provided at least 80% power at 5% level of significance to detect a 20% difference in pancreatic β-cell function, measured as:

$$\beta \;\mathrm{cell}\;\mathrm{insulin}\;\sec\mathrm{retion}=\frac{\mathrm{incremental}\;\mathrm{AUC}\;C-\mathrm{peptide}}{\mathrm{incremental}\;\mathrm{AUC}\;\mathrm{glucose}}\;$$

#### 2.8.2. Demographic, Anthropometric, and Metabolic Data

The statistical environment R was used for all analyses [71], with *p* < 0.05 considered to be statistically significant. The Fisher test was utilised for testing participant distribution by categorical parameters (i.e., sex and ethnicity), while distribution by all other continuous constraints was tested with a paired *t*-test. ANCOVA was utilised to assess the interaction between treatment and time variables, and Tukey’s HSD post-hoc pairwise comparisons were used to compare treatment groups. Pairwise *t*-test comparisons for individual timepoints were conducted using 2-way repeated measures ANOVA. One-way ANOVA was used for iAUC glucose, insulin, C-peptide, and C-peptide/glucose ratio, comparing differences between intervention groups over the 3-month intervention (change from baseline CID 1 to CID 4). iAUC and timepoints plots were expressed as mean ± SEM. At the OGTT, missing single baseline values were calculated using mean baseline values from the other 3 CIDs, whilst multiple missing baseline values were treated as missing data. Missing timepoints mid-OGTT used the average of the time points before and after, assuming linear progression. No final timepoint data were missing for any participant who attended both CID 1 and CID 4.

#### 2.8.3. Gut Microbiota Sequence Data

The statistical environment R was used for all analyses [71], with *p* < 0.05 considered to be statistically significant. A 3-way ANOVA using type 3 sums of squares was applied on the complete dataset to test the response of alpha diversity to the interaction between three key variables (participant treatment, participant health status, time of sample collection). A main-effect ANOVA *p*-value was thus generated, accounting for repeated measures, and paired *t*-tests were also employed to compare alpha diversity over time within cohorts. To construct error bars, 95% confidence intervals were used. Bray-Curtis dissimilarity was utilised for non-metric multidimensional scaling (nMDS) analysis of bacterial community shift in study participants between baseline and the final timepoint, and the significance of change was determined using ANOVA. A permutational analysis of variance (PERMANOVA) was also performed with 1999 unrestricted permutations of raw data, taking into account repeated measures of each participant. Spearman coefficients were used to correlate change in bacterial taxon abundances with change in alpha diversity and clinical measures over time, and resulting *p*-values were false discovery rate-adjusted (FDR).

## 3. Results

### 3.1. Participant Baseline Characteristics and Compliance with Treatment

Eighty-seven (87) participants were enrolled into the study, of which 78 completed the intervention. In turn, 75 participants provided a faecal sample at both baseline CID 1 and end of trial CID 4, with microbiota data obtained from 73 participants due to low DNA yields from 2 samples (Figure 1).

In post-trial plasma glucose batch analyses, 47 participants were identified as normoglycaemic at baseline CID 1 (Control = 15; RC = 12; RY = 20) and 40 participants were identified as prediabetic with raised FPG (Control = 14; RC = 17; RY = 9). FPG differed significantly between these glycaemic sub-groups (mean: 5.1 vs. 5.9 mmol/L; *p* < 0.01). At CID 1 baseline, visceral fat % and glucoregulatory markers fasted insulin (FI) and C-peptide also differed between groups (Table 1).

The subset of 73 participants for whom gut microbiota composition was analysed both pre- and post-intervention were representative of the total 87 enrolled, with similar baseline distribution of anthropometric, body composition, and glucoregulatory measures across sub-groups and treatment cohorts (Supplementary Tables S1–S3). Of the 87 enrolled participants in the full cohort, 23 were of European Caucasian and 64 were of Asian Chinese descent. In this small group of participants, no significant difference was observed at baseline CID 1 between the two ethnic groups for body composition, glucoregulatory markers, LFT, or lipid profile. Hence, we did not further explore ethnicity and participant data were analysed as a single group.

Participant compliance with treatment products was high, with (mean ± SD) 91 ± 14% yoghurt consumption and 90 ± 14% capsule consumption. There was no significant difference in compliance between the 3 treatment groups (*p* > 0.05).

### 3.2. Effect of Rutin Supplementation on Metabolic Health Parameters

**Figure 2:**
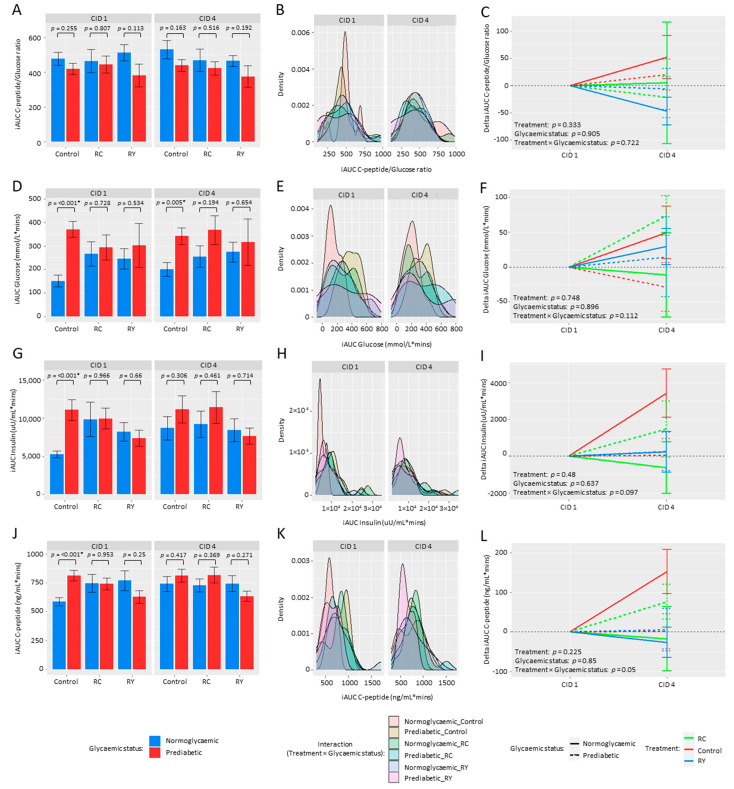
Post-OGTT comparison (mean ± SEM) of iAUC C-peptide/glucose ratio (**A**), iAUC glucose (**D**), iAUC insulin (**G**) and iAUC C-peptide (**J**) between health-associated cohorts for each of the three treatment groups (Control, placebo; RC, rutin capsule; RY, rutin yoghurt). Density plots showing variation in responses to treatment among health-associated cohorts are presented in panels (**B**,**E**,**H**,**K**). Change in respective iAUC parameters over time (CID 1 to CID 4) is presented in plots (**C**,**F**,**I**,**L**) (Mean ± SEM). * *p*-value < 0.05.

In the full cohort, participant body weight was stable across all treatment groups with no significant change over the 12-week duration of the intervention (*p* > 0.05). By chance, baseline body weight was significantly higher in RC versus RY and Control treatments (Mean ± SD; Control: 78 ± 15 kg; RC: 83 ± 15 kg; RY: 77 ± 13 kg; *p* < 0.01). Contrary to our hypothesis, there was no detectable effect of treatment on OGTT glycaemic endpoints, including the primary endpoint iAUC C-peptide/glucose, over 12 weeks, with no significant change in this indirect measure of β-cell insulin secretion over time between RC, RY, or Control treatments (treatment*time, *p* > 0.05). Sub-group analysis of the normoglycaemic and prediabetic participants showed no significant difference in either 120 min OGTT response curves for the primary endpoint C-peptide/glucose or iAUC C-peptide/glucose at either CID 1 or CID 4 in any of RC, RY or Control treatments (Figure 2A–C). Notably, however, there was a trend for lower iAUC C-peptide/glucose ratio in the prediabetic group for all treatments at both CID 1 and CID 4.

Comparison of change in iAUC C-peptide/glucose between CID 1 and CID 4, respectively, also revealed no significant change over 12 weeks for any of the 3 treatments in either participant sub-group (*p* > 0.05, Figure 2C). There were also no significant treatment effects on the secondary metabolic endpoints of iAUC glucose, iAUC insulin, and iAUC C-peptide when analysed as the full cohort and also as glycaemic sub-cohorts (Figure 2F,I,L).

### 3.3. Effect of Rutin Supplementation on Gut Microbiota Composition

**Figure 3:**
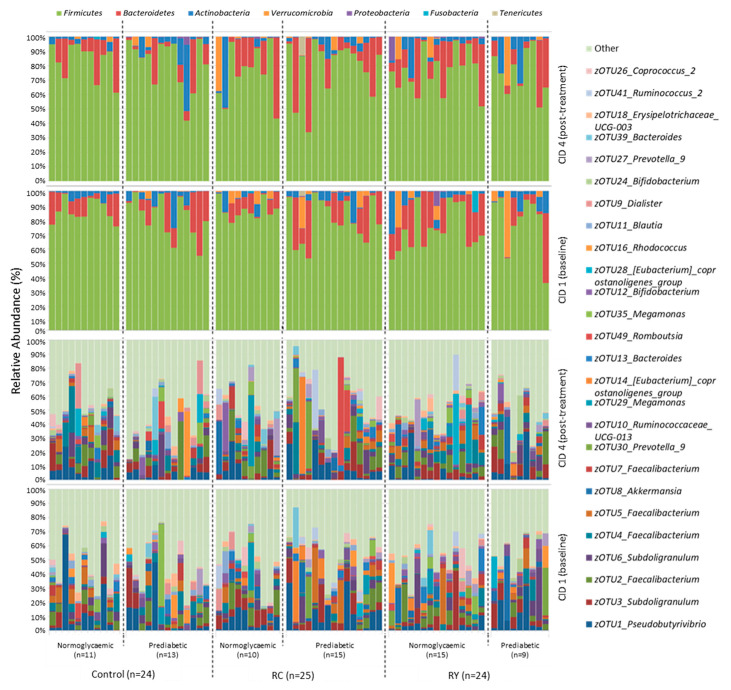
Phylum-level (**top**) and zOTU-level (**bottom**) gut bacterial community composition for all participants investigated from each study arm at CID 1 and CID 4. Each column on the graph represents data from a single participant. zOTUs with ≥1% overall 16S rRNA gene relative sequence abundance are shown, with all remaining zOTUs grouped together in “Other”. Control, placebo; RC, rutin capsule; RY, rutin yoghurt.

Irrespective of glycaemic status or treatment group, *Firmicutes* (79.2 ± 22.4% relative sequence abundance (mean ± S.D.)) was the dominant bacterial phylum in virtually all analysed faecal samples, with *Bacteroidetes* (13.6 ± 11.3%) next most abundant (Figure 3). Some individuals also displayed substantial amounts of *Actinobacteria* (4.8 ± 6.1%) or *Verrucomicrobia* (2.2 ± 5.6%), though these did not correlate with treatment group or glycaemic status. Overall, phylum-level microbiota profiles for individuals were relatively consistent between CID 1 and CID 4, although there were some exceptions. There was considerable inter-individual variation in the microbiota at the zOTU level, with members of the bacterial genera *Pseudobutyrivibrio*, *Subdoligranulum* and *Faecalibacterium* among the most abundant overall (Figure 3).

The ordination (nMDS) plot (Figure S1) further illustrates a notable lack of predictable change in microbiota composition between CID 1 and CID 4. The plot presents vectors from each of the treatment groups featuring varying distances and trajectories that are seemingly independent of treatment (*p* > 0.05).

In an attempt to quantify underlying sources of variation in the microbiota, we used PERMANOVA (taking into account repeated measures due to the multiple time points in our study) (Supplementary Table S6), with neither treatment nor participant glycaemic status significantly affecting microbiota composition. Similarly, there was no significant effect of either treatment or glycaemic status on bacterial alpha diversity metrics (zOTU richness and Shannon diversity) (Supplementary Figure S2A,B).

To identify associations between key metabolic outcomes and specific bacterial taxa, we undertook a Spearman correlation analysis, with significant correlations shown in Table 2. In particular, change in the abundance of *Ruminococcus torques* was negatively associated with the significantly increased FI in the normoglycaemic Control sub-group (Supplementary Figure S3C) (Spearman correlation coefficient = −0.94, *p* < 0.01), and the butyrate producer *Roseburia inulinivorans* was negatively associated with FPG in the prediabetic Control sub-group (Spearman correlation coefficient = −0.86, *p* < 0.03).

## 4. Discussion

As a significant lifestyle-related health problem, T2D is the subject of intensive research into potential dietary treatments, such as plant-derived polyphenols. Previous animal studies have shown that oral rutin treatment can exhibit anti-diabetic properties, with capacity to prevent pancreatic islet human Amylin aggregation, improving both glycaemic control and survival [21,31,72,73,74,75]. Potential mechanisms of action proposed for rutin include decreased carbohydrate absorption in the small intestine, as well as stimulation of β-cell insulin secretion, increased insulin-driven glucose uptake by tissues, and inhibition of hepatic gluconeogenesis [76]. Promising results from human Amylin transgenic mice, in which rutin delivered orally in drinking water delayed development of T2D [21,31], led us to test the effect of rutin supplementation on a cohort of overweight normoglycaemic and prediabetic individuals. Specifically, we explored the effect of flavonoid rutin at a dose of 500 mg/day, on a marker of pancreatic β-cell function and also gut microbiota profiles in a 12-week, double-blind RCT. A secondary objective of this study was to determine whether delivery format was important, and hence, rutin was delivered both macro-encapsulated as rutin powder and micro-encapsulated within a dairy yoghurt food product.

The rutin intervention study presented here is the first human clinical study to investigate postprandial T2D-related blood markers in response to dietary rutin supplementation. In addition to the robust study design, this RCT benefitted from low participant drop-out and good compliance, with more than 90% consumption of both capsule and yoghurt test products over 3 months. Moreover, participant body weight was stable, suggesting that participants adhered to the study guidelines of maintenance of habitual daily dietary and lifestyle patterns.

### 4.1. Rutin Supplementation and Metabolic Health Parameters

The primary hypothesis of this study was that rutin supplementation would increase postprandial iAUC C-peptide/glucose ratio (as an estimate of insulin secretion and β-cell function), insulin and C-peptide, as well as decrease postprandial plasma glucose response to an OGTT. Contrary to this hypothesis, our results showed no detectable improvement in pancreatic β-cell function or other T2D-related blood markers including fasting and postprandial plasma glucose, insulin, and C-peptide, in either normoglycaemic or prediabetic individuals. This was somewhat unexpected given prior evidence of rutin efficacy in T2D rodent models [21,31,72,73,74,75], and we discuss possible reasons for this below. Moreover, we saw no evidence that at-risk individuals with prediabetes showed better improvement in β-cell function, insulin secretion, and glycaemic control compared to the normoglycaemic Control sub-group.

Prior to this trial, very few clinical studies had investigated beneficial effects of rutin supplementation on T2D treatment or prevention. A non-randomised, uncontrolled before-and-after study published in 2011 reported a significant decrease in FPG after 2 months of rutin supplementation in T2D patients, also using a 500 mg/day dose [33]. However, the study lacked a control group, measured only FPG and not postprandial glucose, insulin, or C-peptide levels. More recently, Ragheb et al. (2020) reported decreased FPG in T2D patients given a combination treatment of rutin (180 mg/day) plus vitamin C (480 mg/day) in a 3-treatment, 8-week RCT [77]. However, the vitamin C-only (500 mg/day) cohort also exhibited significantly decreased FPG. In our current 3-treatment RCT, where we advanced these prior trials through investigation of both fasting and OGTT-driven postprandial glycaemic response in overweight but non-T2D participants, we might hypothesise that the lack of glycaemic improvement was due to the absence of significant baseline pancreatic β-cell impairment in the cohort [78]. Pancreatic β-cell dysfunction may occur as a result of ectopic fat deposition within the pancreas [79], elevating free fatty acids (FFA) as well as driving insulin resistance and loss of glycaemic control [80]. As β-cells become overtaxed through increased insulin secretion, they also experience lipotoxicity due to prolonged exposure to FFAs, eventually triggering β-cell dysfunction and decreased insulin secretion [81]. β-cell dysfunction and decreased insulin secretion therefore may not appear until the later stages of prediabetes. With consideration of the C-peptide/glucose ratio of 0.87 proposed as the threshold for insulin treatment by Saisho [46], the participants in our trial exhibited a far higher mean baseline ratio of 2.3 in the normoglycaemic and 2.4 in the prediabetic cohort, hence, displaying very limited impairment of β-cell function. We therefore speculate that beneficial effects of rutin on metabolic health, if any, would be more likely to support long-term maintenance of β-cell function in T2D and be less pronounced in early-stage prediabetes.

### 4.2. Rutin Supplementation and the Gut Microbiota

An additional hypothesis in our current RCT was that dietary rutin would alter the gut bacterial community. However, our results show oral dietary rutin treatment to have a negligible effect on microbiota structure, whether delivered within a food matrix or as an encapsulated powder. In a recent in vitro study, Riva et al. (2020) utilised BONCAT and FACS approaches to identify gut bacteria affected by rutin inclusion in post-anaerobic incubations and observed significant enrichment of members from *Lachnospiraceae*, *Enterobacteriaceae*, *Tannerellaceae,* and *Erysipelotrichaceae* families [43]. However, the faecal samples utilised in their study were obtained from healthy individuals and a much higher rutin dosage than our 500 mg/day was used in their anaerobic incubations (~1.5 g per 5 mL reaction). They also reported high variability in the capacity of individuals to break down rutin, consistent with the high inter-individual microbiota variation we observed in our in vivo analyses. We also observed some apparent variation in the responses to treatment among our cohorts, as seen in the bimodal distributions for some of the density plots shown in Figure 2. Unfortunately, our attempts to compare the microbiotas of the individuals represented by each peak of a bimodal plot (signifying putative responders vs. non-responders) were unsuccessful due to the prohibitively low sample sizes once cohorts were divided so many ways.

Phylum-level taxonomic ratios, between *Firmicutes* and *Bacteroidetes* in particular, have long garnered the attention of gut microbiome researchers. Although reported findings have not always been concordant [82,83,84], especially at deeper taxonomic classifications, bacterial community composition at the phylum level is still considered highly relevant [85]. Our findings show a high relative proportion of *Firmicutes* in comparison to *Bacteroidetes*. In this overweight cohort, our results are consistent with some key landmark studies which observed a similar phylum distribution in overweight/obese cohorts [86,87]. More recent large-scale studies on the Dutch population have also presented similar phylum distributions [88,89]. However, the absence of a lean cohort in our study means we cannot be sure whether this effect is limited to participants with a high BMI. In our current study, some key zOTUs observed with at least 1% overall 16S rRNA gene relative sequence abundance include bacteria often reported to be downregulated in T2D such as *Faecalibacterium* (4 spp.) [90,91,92,93], *Akkermansia* [91,94,95], *Bifidobacterium* (2 spp.) [96,97,98,99], and *Bacteroides* (2 spp.) [91,96,100,101], as well as bacteria reported to be enriched in T2D, such as *Ruminococcus* [91,92,96,102] and *Blautia* [91,100,103,104]. Although it is unclear why an increase in FI levels was observed among the normoglycaemic Control sub-group, Spearman correlation analyses indicate that the change may be linked to decreased abundance of *Ruminococcus torques*. Additionally, higher circulating FPG in prediabetic individuals may correlate with a lower abundance of butyrate-producing *Roseburia inulinivorans*, which is known for its anti-inflammatory capacity [40,105].

The current trial was conducted in accordance with International RCT guidelines; however, several limitations should be noted. A cohort of participants with confirmed prediabetes at recruitment reverted to normoglycaemia by CID 1, decreasing the cohort size and therefore statistical power of the intervention. This difference was likely due to variance in glucose analysis methods and/or physiological normalisation of FPG between screening and CID 1. In addition, the efficacy of the intervention may be detectable only in T2D cohorts; hence, rutin may be useful for treatment but not prevention of diabetes. This was a single-dose RCT, and 500 mg/day rutin, previously shown to be efficacious in preclinical studies and safely tolerated by humans, may have been insufficient to elicit an effect. Additionally, 12 weeks may not have been sufficient duration for the metabolic effects of rutin treatment to emerge to a suitably detectable level, and longer intervention periods may improve the strength of metabolic and microbial response. Finally, this study was designed as a “free-living” clinical trial where participants were free to continue with their daily lifestyle alongside the once-daily rutin treatment. As such, changes in background diet and daily activities were not accounted for, and maintenance of similar lifestyle patterns cannot be assumed.

## 5. Conclusions

In this 12-week, double-blind RCT, unexpectedly, there was no significant effect of oral consumption of rutin at a dose of 500 mg/day, administered either as an encapsulated powder or in a food matrix, on postprandial T2D-related blood markers (as an indirect measure of pancreatic β-cell function) in overweight normo- and dysglycaemic individuals. However, this may be due to participants being in the early stages of prediabetes and maintaining unimpaired β-cell function. We also did not observe a significant impact on overall gut microbiota composition as a consequence of this dietary intervention. A high relative proportion of *Firmicutes* in comparison to *Bacteroidetes* was observed irrespective of glycaemic status or treatment, and a negative association between circulating FPG and the abundance of butyrate-producing *Roseburia inulinivorans*, which is known for its anti-inflammatory capacity [40,105], was identified in individuals with prediabetes.

## Figures and Tables

**Table 1:** Baseline characteristics of enrolled and randomized participants.

	All (n = 87)	Normoglycaemic (n = 47)	Prediabetic (n = 40)	*p*-Value
Sex (M:F)	39:48	17:30	22:18	0.088
Ethnicity (C:A)	23:64	11:36	12:28	0.626
Age (years)	44.3 (21–64)	42.7 (22–64)	46.2 (21–64)	0.182
Body weight (kg)	79.5 (54.3–124.2)	77.9 (54.3–105.3)	81.3 (56.1–124.2)	0.293
Height (m)	1.7 (1.4–1.9)	1.7 (1.4–1.9)	1.7 (1.5–1.9)	0.386
Body mass index, BMI (kg/m^2^)	27.6 (22.1–37.8)	27.4 (22.1–37.8)	27.9 (22.3–35.8)	0.537
Waist circumference (cm)	93.9 (73–122)	92.2 (73–112)	95.8 (81–122)	0.102
Hip circumference (cm)	104.3 (83.5–127)	104.5 (88–127)	104.1 (83.5–125)	0.8
Systolic blood pressure (mmHg)	120.4 (91–167)	119.4 (91–157)	121.5 (91–167)	0.562
Diastolic blood pressure (mmHg)	65.3 (47–101)	64.6 (50–101)	66.1 (47–89.7)	0.526
Body Composition				
Total body fat (%)	36.4 (19–52.1)	37.4 (19–52.1)	35.2 (21.9–50.2)	0.188
Abdominal fat (%)	42.6 (18.4–60.6)	43.1 (18.4–60.5)	42.1 (19.9–60.6)	0.617
Visceral fat (%)	42.9 (2.3–90.6)	39.4 (2.3–72.9)	47.2 (7.6–90.6)	0.014 *
Subcutaneous fat (%)	57.1 (9.4–97.7)	60.6 (27.1–97.7)	52.8 (9.4–92.4)	0.014 *
Glycaemic, Liver Function, Lipid biomarkers				
Fasting plasma glucose, FPG (mmol/L)	5.5 (4.5–6.7)	5.1 (4.5–5.5)	5.9 (5.6–6.7)	<0.001 *
Fasting insulin (uU/mL)	12 (2.3–42.6)	10.6 (2.3–42.6)	13.6 (4.69–32.4)	0.03 *
Fasting C-peptide (ng/mL)	2.3 (0.8–4.9)	2.1 (0.8–4.9)	2.6 (1.4–4.3)	0.016 *
Fasting C-peptide (ng/mL)/FPG (mg/dL) ratio (×100)	2.3 (1–5.2)	2.3 (1–5.2)	2.4 (1.3–3.9)	0.552
Alanine aminotransferase, ALT (U/L)	14.1 (2.7–51.9)	12.8 (2.7–48.6)	15.6 (4.1–51.9)	0.197
Aspartate aminotransferase, AST (U/L)	23.4 (11.9–111.2)	23.8 (12.9–97.5)	23.1 (11.9–111.2)	0.834
Alkaline phosphatase, ALP (U/L)	65.4 (35–120)	63.4 (35–110)	67.8 (36–120)	0.279
Gamma-glutamyl transferase, GGT (U/L)	26.4 (5–162)	24.9 (5–162)	28.1 (8–108)	0.581
Total cholesterol (mmol/L)	4.9 (2.3–7.6)	5.0 (3.4–7.6)	4.7 (2.3–6.5)	0.093
HDL-C (mmol/L)	1.2 (0.7–2)	1.3 (0.7–2)	1.2 (0.7–1.9)	0.327
LDL-C (mmol/L)	3 (0.9–5.9)	3.1 (1.9–5.9)	2.9 (0.9–4.3)	0.124
Triglyceride (mmol/L)	1.6 (0.5–7.6)	1.5 (0.5–7.6)	1.6 (0.6–5.3)	0.767

Male, M; female, F; Caucasian, C; Asian, A. Data presented as mean (range) for all other variables. Abdominal fat (%) is presented as proportion of total abdominal mass (g), and visceral fat (%) as proportion of total abdominal fat mass (g). *p*-Values represent difference in distribution between normoglycaemic and prediabetic groups. Fisher test was utilised for sex and ethnicity categorical variables, and paired *t*-tests for all other continuous constraints. * *p*-value < 0.05.

**Table 2:** Spearman coefficient correlations presenting significantly associated gut bacterial taxa with change in bacterial richness (number of observed zOTUs), Shannon diversity, fasting plasma glucose (FPG) and fasting insulin (FI) over time.

	Variable	Treatment Group	zOTUs Identified	Spearman Coefficient	*p*-Value
					(FDR-adj.)
Normoglycaemic cohort	Δ Bacterial richness	Control	*Otu32_Bacteroidetes_Bacteroides*	0.890	0.039 *
		RY	*Otu79_Bacteroidetes_Bacteroides_Bacteroides_sp_’Smarlab_BioMol-2301151′*	0.810	0.041 *
	Δ Shannon diversity	Control	*Otu154_Firmicutes_[Eubacterium]_hallii_groups:butyrate-producing_bacterium_SL6/1/1*	0.914	0.013 *
	Δ FI	Control	*Otu67_Firmicutes_Lachnoclostridium:[Ruminococcus]_torques*	−0.936	0.004 *
Prediabetic cohort	Δ FPG	Control	*Otu34_Firmicutes_Roseburia_Roseburia_inulinivorans*	−0.861	0.025 *

Control, placebo; RC, rutin capsule; RY, rutin yoghurt. * *p*-value < 0.05.

## Data Availability

The 16S rRNA gene sequence data were deposited in Genbank under SRA Bioproject PRJNA946170.

## Supplementary Materials

The following supporting information can be downloaded at: https://www.mdpi.com/article/10.3390/nu15153360/s1, Table S1. Baseline demographic, anthropometric, metabolic and body composition information for the sub-group of microbiome participants, distributed by health-associated cohorts. Table S2. Baseline demographic, anthropometric, metabolic and body composition information for all enrolled participants, distributed by intervention cohorts. Table S3. Baseline demographic, anthropometric, metabolic and body composition information for the sub-group of microbiota participants, distributed by intervention cohorts. Table S4. Formulation of the capsule treatments. Table S5. Energy and macronutrient composition of the yoghurt treatments. Table S6. Results from permutational analysis of variance (PERMANOVA) with consideration for repeated measures, to test significance of bacterial community variation. Figure S1. Visualisation of bacterial community beta diversity. (A) Non-metric multidimensional scaling (nMDS) of the bacterial community shift in study participants between baseline to final time point, based on Bray-Curtis dissimilarity. Vector arrows link baseline time point to final time point. Length of arrow represents the magnitude of change occurring. (B) Box and whisker plot comparing Bray-Curtis distances for each treatment group. Control, placebo; RC, rutin capsule; RY, rutin yoghurt. Figure S2. Mean alpha diversity parameters compared over time (CID 1, red symbol; CID 4, blue symbol) in health-associated cohorts (N, normoglycaemic; P, prediabetic), as distributed by treatment groups (RC, rutin capsule; Control; RY, rutin yoghurt), in sub-group of microbiome participants (n = 73). Error bars, 95% confidence intervals. *p*-Values determined using pairwise *t*-tests to account for repeated measures; main-effect ANOVA *p*-value presents the interaction between health status and treatment over time, in response to the variable tested. (A) observed zOTUs (B) log2_Shannon diversity index (C) Jost diversity index of order 1 (D) Simpson diversity index (E) Berger-Parker diversity index. Figure S3. Mean clinical parameters compared over time (CID 1, red symbol; CID 4, blue symbol) in health-associated cohorts (N, normoglycaemic; P, prediabetic), as distributed by treatment groups (RC, rutin capsule; Control; RY, rutin yoghurt), in sub-group of microbiome participants (n = 73). Error bars, 95% confidence intervals. *p*-Values determined using pairwise *t*-tests to account for repeated measures; main-effect ANOVA *p*-value presents the interaction between health status and treatment over time, in response to the variable tested. (A) fasting plasma glucose, FPG (mmol/L) (B) body mass index, BMI (kg/m^2^) (C) fasting insulin (uU/mL) (D) fasting C-peptide (ng/mL) (E) fasting C-peptide (ng/mL)/FPG (mmol/L) ratio.
