# Supplementary material for: Effect of a 12-Week Polyphenol Rutin Intervention on Markers of Pancreatic β-Cell Function and Gut Microbiota in Adults with Overweight without Diabetes
Source: Nutrients. 2023 Jul 28;15(15):3360. doi: 10.3390/nu15153360 (PMC10420824; doi:10.3390/nu15153360)
Supplement: Supplementary file 1 [file nutrients-15-03360-s001.zip › nutrients-2510729-supplementary.pdf]

Supplementary Table S1. Baseline demographic, anthropometric, metabolic and body composition information for the sub-group of microbiome participants, distributed by health-associated cohorts.

	**All (n = 73)**	**Normoglycaemic (n = 36)**	**Prediabetic (n = 37)**	***p*-Value**
Sex (M:F)	33:40	12:24	21:16	0.061
Ethnicity (C:A)	21:52	9:27	12:25	0.61
Age (years)	45.7 (21-64)	44.8 (23-64)	46.5 (21-64)	0.551
Bodyweight (kg)	79.3 (54.3-124.2)	76.7 (54.3-105.3)	81.8 (56.1-124.2)	0.148
Height (m)	1.7 (1.4-1.9)	1.7 (1.4-1.9)	1.7 (1.5-1.9)	0.134
Body Mass Index, BMI (kg/m^2)	27.6 (22.1-37.8)	27.3 (22.1-37.8)	27.9 (22.3-35.8)	0.477
Waist circumference (cm)	93.7 (73-122)	91.5 (73-112)	95.8 (81-122)	0.068
Hip circumference (cm)	104.2 (88-127)	104.1 (88-127)	104.3 (89-125)	0.95
Systolic blood pressure, SBP (mmHg)	121.9 (91-167)	121.3 (91-157)	122.4 (91-167)	0.783
Diastolic blood pressure, DBP (mmHg)	65.9 (47-101)	65.5 (50-101)	66.2 (47-89.7)	0.791
**Body Composition**				
Total body fat, TBF (%)	35.5 (19-51.8)	37.1 (19-51.8)	34.1 (21.9-50.2)	0.258
Abdominal fat (%)	41.6 (18.4-60.6)	42.5 (18.4-60.5)	40.7 (19.9-60.6)	0.762
Visceral fat (%)	42.8 (2.3-90.6)	39.1 (2.3-72.9)	46.3 (7.6-90.6)	0.021 *
Subcutaneous fat (%)	57.2 (9.4-97.7)	60.9 (27.1-97.7)	53.7 (9.4-92.4)	0.021 *
**Glycaemic, Liver Function, Lipid biomarkers**				
Fasting plasma glucose, FPG (mmol/L)	5.6 (4.6-6.7)	5.2 (4.6-5.5)	5.9 (5.6-6.7)	<0.001 *
Fasting insulin (uU/mL)	11.5 (2.3-32.4)	9.6 (2.3-23.1)	13.3 (4.7-32.4)	0.004 *
Fasting C-peptide (ng/mL)	2.3 (0.8-4.3)	2.1 (0.8-3.8)	2.6 (1.4-4.3)	0.009 *
Fasting C-peptide (ng/mL)/FPG (mg/dL) ratio (x100)	2.3 (1-3.9)	2.2 (1-3.8)	2.4 (1.3-3.9)	0.349
Alanine aminotransferase, ALT (U/L)	13.7 (2.7-51.9)	12.3 (2.7-48.6)	15 (4.1-51.9)	0.221
Aspartate aminotransferase, AST (U/L)	23.6 (11.9-111.2)	24.2 (13.1-97.5)	23 (11.9-111.2)	0.748
Alkaline phosphatase, ALP (U/L)	65.8 (35-120)	63.9 (35-110)	67.6 (36-120)	0.406
Gamma-glutamyl transferase, GGT (U/L)	27.1 (5-162)	25.8 (5-162)	28.4 (8-108)	0.493
Total cholesterol (mmol/L)	4.9 (2.3-7.6)	5.2 (3.4-7.6)	4.7 (2.3-6)	0.016 *
HDL-cholesterol (mmol/L)	1.2 (0.7-2)	1.3 (0.7-2)	1.2 (0.8-1.8)	0.073
LDL-cholesterol (mmol/L)	3 (0.9-5.9)	3.2 (1.9-5.9)	2.9 (0.9-4.2)	0.044 *
Triglycerides (mmol/L)	1.6 (0.5-7.6)	1.6 (0.5-7.6)	1.6 (0.6-5.3)	0.848

Male, M; female, F; Caucasian, C; Asian, A. Data presented as mean (range) for all other variables. Abdominal fat (%) is presented as proportion of total abdominal mass (g), and visceral fat (%) as proportion of total abdominal fat mass (g). *p*-Values represent difference in distribution between normoglycaemic and prediabetic groups. Fisher test was utilised for sex and ethnicity categorical variables, and paired *t*-tests for all other continuous constraints. * p-value < 0.05.

Supplementary Table S2. Baseline demographic, anthropometric, metabolic and body composition information for all enrolled participants, distributed by intervention cohorts.

	**All (n = 87)**	**Control (n = 29)**	**Rutin Capsule (n = 29)**	**Rutin Yoghurt (n = 29)**	***p*-value**	
Sex (M:F)	39:48	12:17	15:14	12:17	0.766	
Ethnicity (C:A)	23:64	6:23	10:19	7:22	0.561	
Age (years)	44.3 (21-64)	45.8 (23-64)	46.3 (29-64)	40.8 (21-63)	0.157	
Bodyweight (kg)	79.5 (54.3-124.2)	78.2 (56.1-124.2)	83.4 (56.2-111.5)	76.9 (54.3-104.9)	0.222	
Height (m)	1.7 (1.4-1.9)	1.7 (1.5-1.9)	1.7 (1.5-1.9)	1.7 (1.4-1.9)	0.437	
Body Mass Index, BMI (kg/m^2)	27.6 (22.1-37.8)	26.9 (22.3-37.8)	28.5 (22.1-35.8)	27.4 (22.7-36.2)	0.259	
Waist circumference (cm)	93.9 (73-122)	93 (80-122)	96.9 (78-119)	91.7 (73-112)	0.123	
Hip circumference (cm)	104.3 (83.5-127)	104.2 (88-126)	105.7 (83.5-127)	103.1 (90-125.1)	0.545	
Systolic blood pressure, SBP (mmHg)	120.4 (91-167)	117.5 (91-167)	124.4 (91-161)	119.3 (96-156)	0.268	
Diastolic blood pressure, DBP (mmHg)	65.3 (47-101)	62.4 (47-79)	69.4 (54-89.7)	64.2 (53-101)	0.040	*
**Body Composition**						
Total body fat, TBF (%)	36 (19-52.1)	35.9 (26.7-51.8)	36.2 (23.8-50.7)	35.8 (19-52.1)	0.775	
Abdominal fat (%)	42.1 (18.4-60.6)	42 (31.7-60.5)	43.3 (24.8-57.7)	41.2 (18.4-60.6)	0.59	
Visceral fat (%)	42.4 (2.3-90.6)	40.3 (19.4-62.9)	46.9 (2.3-90.6)	40.1 (20.3-65.3)	0.187	
Subcutaneous fat (%)	57.6 (9.4-97.7)	59.7 (37.1-80.6)	53.1 (9.4-97.7)	59.9 (34.7-79.7)	0.187	
**Glycaemic, Liver Function, Lipid biomarkers**						
Fasting plasma glucose, FPG (mmol/L)	5.5 (4.5-6.7)	5.4 (4.5-6.2)	5.6 (4.7-6.7)	5.5 (4.7-6.7)	0.588	
Fasting insulin (uU/mL)	12 (2.3-42.6)	10.6 (2.3-21.5)	12.7 (3.1-32.4)	12.7 (4.7-42.6)	0.375	
Fasting C-peptide (ng/mL)	2.3 (0.8-4.9)	2.2 (1-3.7)	2.6 (0.8-4.3)	2.3 (1.2-4.9)	0.138	
Fasting C-peptide/FPG (mg/dL) ratio (x100)	2.3 (1-5.2)	2.2 (1.1-3.5)	2.6 (1-3.9)	2.3 (1.3-5.2)	0.159	
Alanine aminotransferase, ALT (U/L)	14.1 (2.7-51.9)	12.6 (3-35)	15.5 (4.1-51.9)	14.2 (2.7-48.6)	0.558	
Aspartate aminotransferase, AST (U/L)	23.4 (11.9-111.2)	23.4 (11.9-97.5)	23.2 (13.1-73.1)	23.7 (13.4-111.2)	0.994	
Alkaline phosphatase, ALP (U/L)	65.4 (35-120)	64 (35-120)	63.7 (36-111)	68.6 (37-110)	0.523	
Gamma-glutamyl transferase, GGT (U/L)	26.4 (5-162)	20.9 (7-83)	31.7 (9-108)	26.6 (5-162)	0.288	
Total cholesterol (mmol/L)	4.9 (2.3-7.6)	4.8 (3.4-6.2)	5.1 (2.3-6.9)	4.8 (2.8-7.6)	0.378	
HDL-cholesterol (mmol/L)	1.2 (0.7-2)	1.2 (0.7-1.8)	1.2 (0.8-1.9)	1.3 (0.7-2)	0.327	
LDL-cholesterol (mmol/L)	3 (0.9-5.9)	3 (1.9-4.2)	3.2 (1-4.3)	2.9 (0.9-5.9)	0.402	
Triglycerides (mmol/L)	1.6 (0.5-7.6)	1.4 (0.6-2.8)	1.7 (0.5-5.3)	1.6 (0.6-7.6)	0.532	

Male, M; female, F; Caucasian, C; Asian, A. Data presented as mean (range) for all other variables. Abdominal fat (%) is presented as proportion of total abdominal mass (g), and visceral fat (%) as proportion of total abdominal fat mass (g). *p*-Values represent difference in distribution between normoglycaemic and prediabetic groups. Fisher test was utilised for sex and ethnicity categorical variables, and paired *t*-tests for all other continuous constraints. * p-value < 0.05.

Supplementary Table S3. Baseline demographic, anthropometric, metabolic and body composition information for the sub-group of microbiota participants, distributed by intervention cohorts.

	All (n = 73)	Control (n = 24)	Rutin Capsule (n = 25)	Rutin Yoghurt (n = 24)	*p*-Value
Sex (M:F)	33:40	10:14	13:12	10:14	0.73
Ethnicity (C:A)	21:52	19:5	10:15	6:18	0.354
Age (years)	45.7 (21-64)	48.6 (26-64)	45.9 (29-62)	42.5 (21-63)	0.202
Body weight (kg)	79.3 (54.3-124.2)	77.8 (56.1-124.2)	84.7 (59.4-111.5)	75.1 (54.3-102.4)	0.07
Height (m)	1.7 (1.4-1.9)	1.7 (1.5-1.9)	1.7 (1.5-1.9)	1.7 (1.4-1.9)	0.465
Body Mass Index, BMI (kg/m^2)	27.6 (22.1-37.8)	27.1 (22.3-37.8)	28.9 (22.1-35.8)	26.8 (22.7-35.8)	0.082
Waist circumference (cm)	93.7 (73-122)	93.2 (80-122)	97.2 (78-119)	90.6 (73-111)	0.069
Hip circumference (cm)	104.2 (88-127)	103.9 (88-126)	106.6 (88-127)	102 (90-121)	0.197
Systolic blood pressure, SBP (mmHg)	121.9 (91-167)	119.5 (91-167)	125.7 (91-161)	120.3 (96-156)	0.402
Diastolic blood pressure, DBP (mmHg)	65.9 (47-101)	63.5 (47-79)	69.8 (54-89.7)	64.2 (53-101)	0.1
**Body Composition**					
Total body fat, TBF (%)	35.5 (19-51.8)	35.6 (26.7-51.8)	36.2 (23.8-50.7)	34.8 (19-50.2)	0.571
Abdominal fat (%)	41.6 (18.4-60.6)	41.8 (31.7-60.5)	43.1 (24.8-57.7)	39.7 (18.4-60.6)	0.325
Visceral fat (%)	42.8 (2.3-90.6)	40.6 (19.4-62.9)	47.2 (2.3-90.6)	40.3 (20.3-60.1)	0.293
Subcutaneous fat (%)	57.2 (9.4-97.7)	59.4 (37.1-80.6)	52.8 (9.4-97.7)	59.7 (39.9-79.7)	0.293
**Glycaemic, Liver Function, Lipid biomarkers**					
Fasting plasma glucose, FPG (mmol/L)	5.6 (4.6-6.7)	5.5 (4.6-6.2)	5.6 (4.8-6.7)	5.6 (4.7-6.7)	0.599
Fasting insulin (uU/mL)	11.5 (2.3-32.4)	10.4 (2.3-21.5)	12.7 (3.1-32.4)	11.4 (4.7-21.8)	0.349
Fasting C-peptide (ng/mL)	2.3 (0.8-4.3)	2.2 (1-3.7)	2.6 (0.8-4.3)	2.1 (1.2-4)	0.093
Fasting C-peptide/FPG (mg/dL) ratio (x100)	2.3 (1-3.9)	2.2 (1.1-3.5)	2.6 (1-3.9)	2.1 (1.3-3.6)	0.088
Alanine aminotransferase, ALT (U/L)	13.7 (2.7-51.9)	12.6 (4.1-35)	14.8 (4.1-51.9)	13.7 (2.7-48.6)	0.713
Aspartate aminotransferase, AST (U/L)	23.6 (11.9-111.2)	23.4 (11.9-97.5)	23.5 (13.1-73.1)	23.9 (13.4-111.2)	0.994
Alkaline phosphatase, ALP (U/L)	65.8 (35-120)	65.6 (35-120)	62.3 (36-111)	69.6 (37-110)	0.411
Gamma-glutamyl transferase, GGT (U/L)	27.1 (5-162)	22.1 (7-83)	31.4 (9-108)	27.6 (5-162)	0.493
Total cholesterol (mmol/L)	4.9 (2.3-7.6)	4.9 (3.4-6.2)	5.1 (2.3-6.9)	4.9 (2.8-7.6)	0.632
HDL-cholesterol (mmol/L)	1.2 (0.7-2)	1.3 (0.9-1.8)	1.2 (0.8-1.8)	1.3 (0.7-2)	0.209
LDL-cholesterol (mmol/L)	3 (0.9-5.9)	3 (1.9-4.2)	3.1 (1-4.2)	2.9 (0.9-5.9)	0.649
Triglycerides (mmol/L)	1.6 (0.5-7.6)	1.4 (0.6-2.8)	1.8 (0.5-5.3)	1.6 (0.6-7.6)	0.526

Male, M; female, F; Caucasian, C; Asian, A. Data presented as mean (range) for all other variables. Abdominal fat (%) is presented as proportion of total abdominal mass (g), and visceral fat (%) as proportion of total abdominal fat mass (g). *p*-values represent difference in distribution between normoglycaemic and prediabetic groups. Fisher test was utilised for sex and ethnicity categorical variables, and paired *t*-tests for all other continuous constraints.

Supplementary Table S4. Formulation of the capsule treatments.

	Rutin Capsule	Placebo Capsule
Serving size	2 capsules	2 capsules
Rutin (mg)	250	0
Maltodextrin (mg)	0	250
Silica dioxide (mg)	0.2	0.2
Magnesium stearate (mg)	0.5	0.5
Microcrystalline cellulose (mg)	99.3	99.3
Total weight (mg)	350	350

Supplementary Table S5. Energy and macronutrient composition of the yoghurt treatments.

	Rutin-Enriched Yoghurt	Placebo Yoghurt
Serving size (g)	190	190
Energy (kJ)	468	474
Protein (g)	9.3	9.2
Fat (g)	3.9	3.9
Carbohydrate (g)	10.4	10.6
Sodium (mg)	85	87
Calcium (mg)	269	274
Rutin (mg)	~500	0

Supplementary Table S6. Results from permutational analysis of variance (PERMANOVA) with consideration for repeated measures, to test significance of bacterial community variation.

			Normoglycaemic (n = 36)		Prediabetic (n = 37)		All (n = 73)		
	n	Variable(s) Tested	R^2	*p*-Value	R^2	*p*-Value	R^2	*p*-Value	
**Treatment**	73	All	0.007	0.721	0.010	0.122	0.005	0.082	
	24	Placebo (Control)	0.031	0.302	0.029	0.069	0.016	0.098	
	25	Capsule (RC)	0.026	0.566	0.018	0.681	0.012	0.396	
	24	Yoghurt (RY)	0.015	0.920	0.022	0.931	0.009	0.886	
	49	Rutin (RC+RY)	0.010	0.727	0.010	0.718	0.007	0.250	
**Gender**	33	Male	0.024	0.328	0.013	0.492	0.009	0.301	
	40	Female	0.011	0.721	0.025	0.084	0.009	0.197	
**Ethnicity**	21	European Caucasian	0.032	0.466	0.020	0.544	0.013	0.372	
	52	Asian Chinese	0.010	0.536	0.017	0.081	0.009	0.031	*

* *p*-value <0.05.

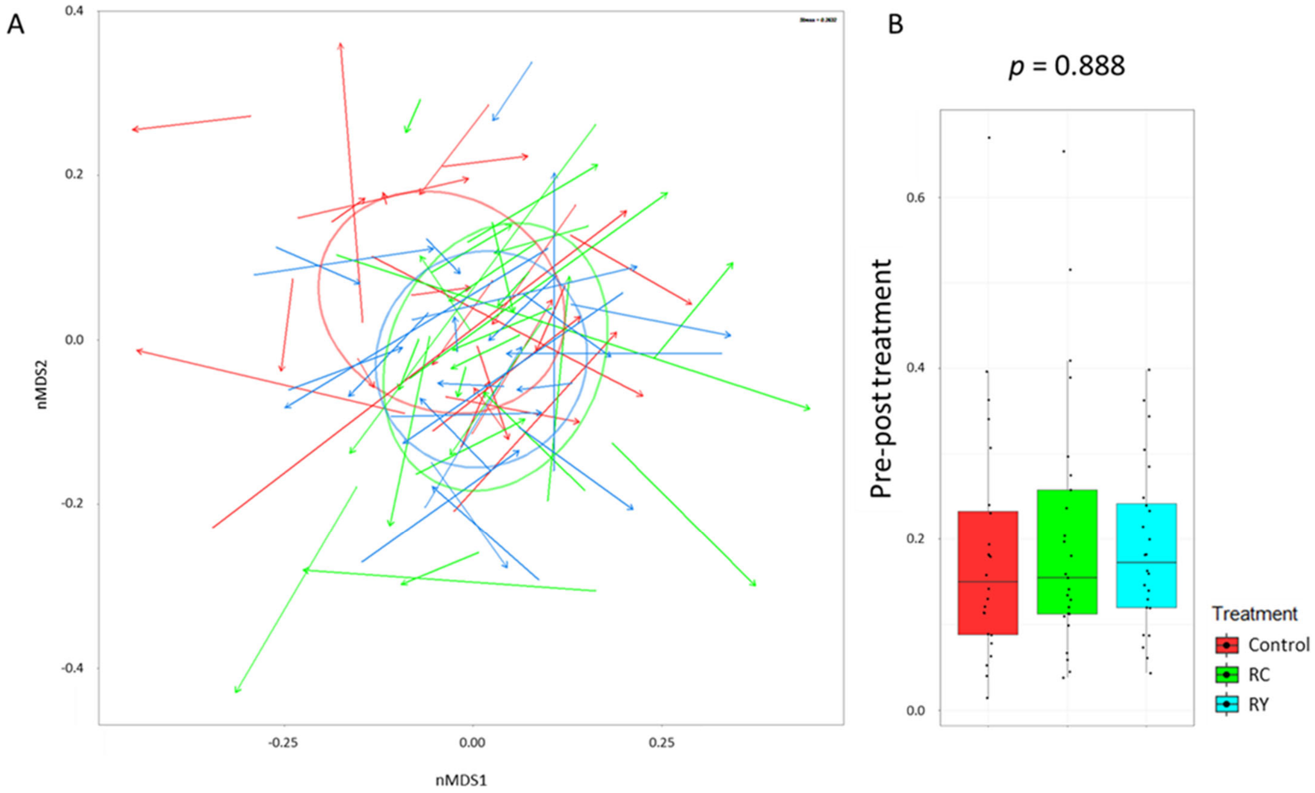

Figure S1. Visualisation of bacterial community beta diversity. (**A**) Non-metric multidimensional scaling (nMDS) of the bacterial community shift in study participants between baseline to final time point, based on Bray-Curtis dissimilarity. Vector arrows link baseline time point to final time point. Length of arrow represents the magnitude of change occurring. (**B**) Box and whisker plot comparing Bray-Curtis distances for each treatment group. Control, placebo; RC, rutin capsule; RY, rutin yoghurt.

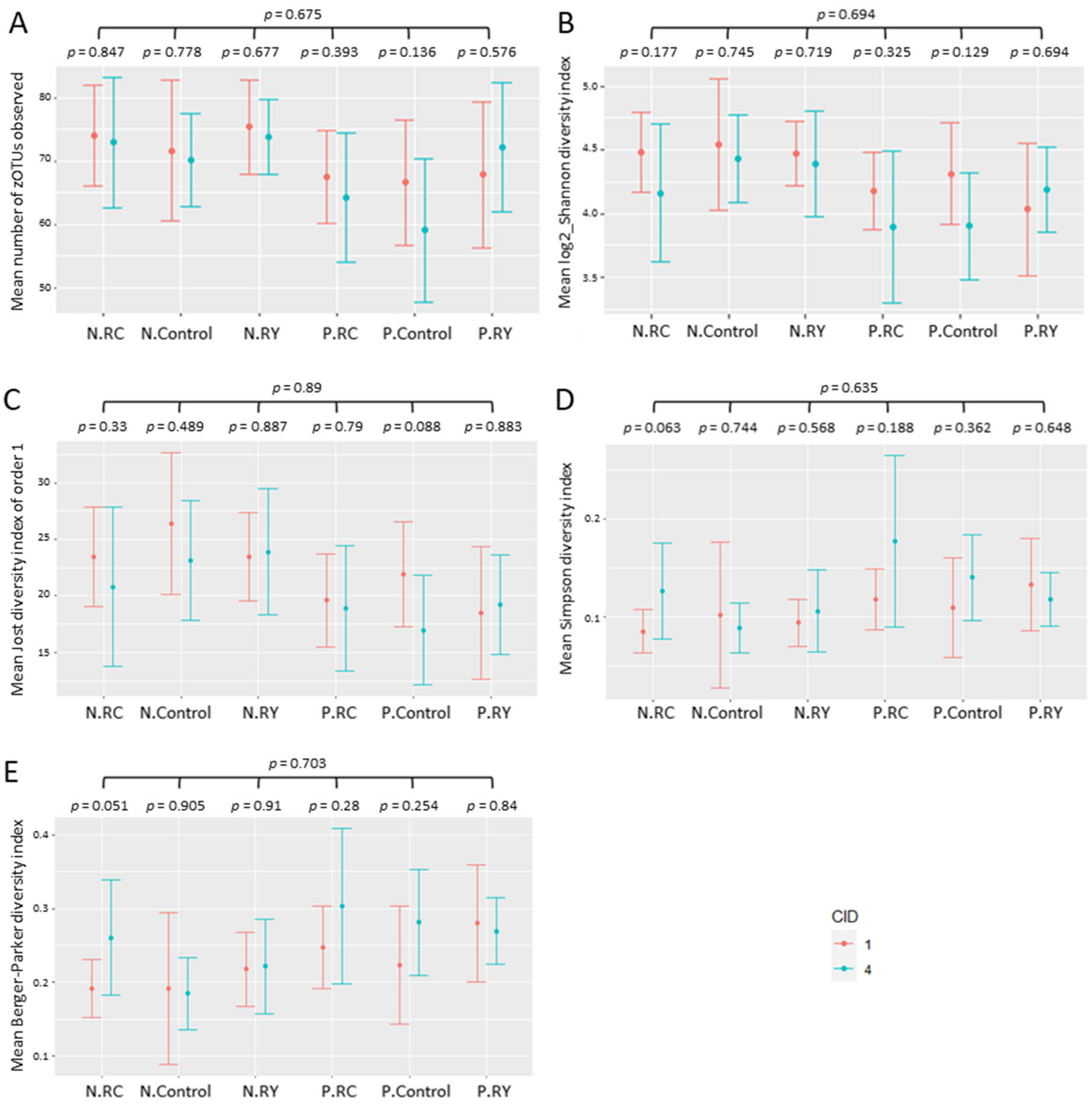

Supplementary Figure S2. Mean alpha diversity parameters compared over time (CID 1, red symbol; CID 4, blue symbol) in health-associated cohorts (N, normoglycaemic; P, prediabetic), as distributed by treatment groups (RC, rutin capsule; Control; RY, rutin yoghurt), in sub-group of microbiome participants (n = 73). Error bars, 95% confidence intervals. *P*-values determined using pairwise t-tests to account for repeated measures; main-effect ANOVA *p*-value presents the interaction between health status and treatment over time, in response to the variable tested. (**A**) observed zOTUs (**B**) log2_Shannon diversity index (**C**) Jost diversity index of order 1 (**D**) Simpson diversity index (**E**) Berger-Parker diversity index.

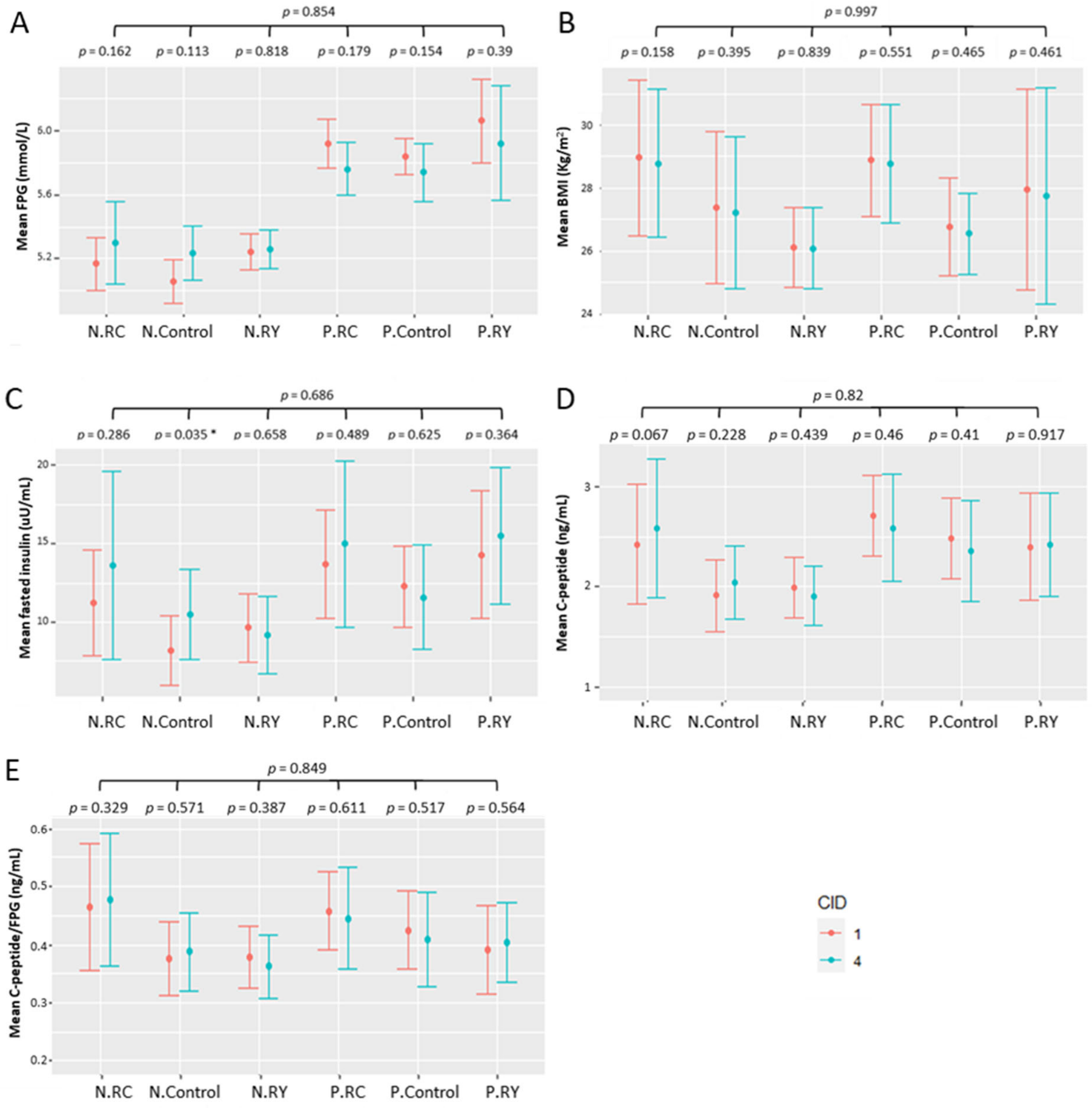

Supplementary Figure S3. Mean clinical parameters compared over time (CID 1, red symbol; CID 4, blue symbol) in health-associated cohorts (N, normoglycaemic; P, prediabetic), as distributed by treatment groups (RC, rutin capsule; Control; RY, rutin yoghurt), in sub-group of microbiome participants (n = 73). Error bars, 95% confidence intervals. *P*-values determined using pairwise t-tests to account for repeated measures; main-effect ANOVA *p*-value presents the interaction between health status and treatment over time, in response to the variable tested. (**A**) fasting plasma glucose, FPG (mmol/L) (**B**) body mass index, BMI (kg/m^2) (**C**) fasting insulin (uU/mL) (**D**) fasting C-peptide (ng/mL) (**E**) fasting C-peptide (ng/mL)/FPG (mmol/L) ratio. * p-value < 0.05.
